# Extracellular vesicles derived from microgreens of *Raphanus sativus* L. var. *caudatus* Alef contain bioactive macromolecules and inhibit HCT116 cells proliferation

**DOI:** 10.1038/s41598-022-19950-7

**Published:** 2022-09-20

**Authors:** Karnchanok Kaimuangpak, Kawintra Tamprasit, Kanjana Thumanu, Natthida Weerapreeyakul

**Affiliations:** 1 Graduate School (in the Program of Research and Development in Pharmaceuticals), Faculty of Pharmaceutical Sciences, Khon Kaen University, Khon Kaen, 40002 Thailand; 2 Research Institute for Human High Performance and Health Promotion, Khon Kaen University, Khon Kaen, 40002 Thailand; 3 Synchrotron Light Research Institute (Public Organization), Nakhon Ratchasima, 30000 Thailand; 4 Division of Pharmaceutical Chemistry, Faculty of Pharmaceutical Sciences, Khon Kaen University, 123 Mittrapap Road, Amphoe Muang, Khon Kaen, 40002 Thailand

**Keywords:** Cancer, Nanomedicine

## Abstract

Extracellular vesicles (EVs) are phospholipid bilayer vesicles released from cells, containing natural cargos. Microgreens of *Raphanus sativus* L. var. *caudatus* Alef were used in this study as the source of EVs. EVs were isolated by differential centrifugation. The physical properties were determined by dynamic light scattering (DLS) and electron microscopy. The biological and chemical composition were studied by Fourier**-**transform infrared (FTIR) microspectroscopy and high-performance liquid chromatography analysis, respectively. EVs had a median size of 227.17 and 234.90 ± 23.30 nm determined by electron microscopy and DLS, respectively with a polydispersity index of 0.293 ± 0.019. Electron microscopy indicated the intact morphology and confirmed the size. The FTIR spectra revealed that EVs are composed of proteins as the most abundant macromolecules. Using a curve-fitting analysis, β-pleated sheets were the predominant secondary structure. Notably, the micromolecular biomarkers were not detected. EVs exerted anti-cancer activity on HCT116 colon cancer over Vero normal cells with an IC_50_ of 448.98 µg/ml and a selectivity index of > 2.23. To conclude, EVs could be successfully prepared with a simple and effective isolation method to contain nano-sized macromolecules possessing anti-cancer activity.

## Introduction

Plants are a natural source of secondary metabolites possessing various biological activities. Plant secondary metabolites are specific for a few plant genera or families as they are synthesized with specific physiological and ecological functions through their adaptation to growth environment and stress. Global warming and climate change have already disrupted and limited agricultural supply^1^. Global warming and resultant climate change caused by increases in atmospheric carbon dioxide are having an impact on the production and amounts of secondary metabolites^2^. In light of the negative impact on sustainability, availability, and sufficiency of active compounds under global warming, seedlings (sprouts and microgreens) could represent a new source of nutrients and bioactive compounds^3^. Importantly, microgreens can be planted throughout the year, grow rapidly, and are mass-producible. Seedlings of cruciferous vegetables in the Brassicaceae family contain numerous bioactive constituents, including phenolic compounds, glucosinolates^4,5^, isothiocyanates^4^, anthocyanins, phenylpropanoids, and carotenoids^5^. Both pure compounds and extracts from seedlings of cruciferous vegetables show promising bioactivity, including anti-cancer^6^, anti-inflammation^7^, and anti-oxidation^8^. In this study, we used Thai rat-tailed radish microgreens—a vegetable in the Brassicaceae family—as a source to isolate extracellular vesicles (EVs).

Thai rat-tailed radish (Khi Hood in Thai) or *Raphanus sativus* L. var. *caudatus* Alef belongs to the Brassicaceae family. Phenolic compounds^9–11^, glucosinolates, and isothiocyanates, particularly sulforaphene and sulforaphane^11–15^, can be isolated from different parts of Thai rat-tailed radish. These phytochemical bioactive compounds confer antioxidant properties^9,10^, tyrosinase inhibitory activity^11^, and anti-cancer activity; evidenced by (a) antiproliferation of three different cell lines (colorectal carcinoma HCT116, breast cancer MCF-7, and lung adenocarcinoma SK-LU1 cell lines), and (b) an apoptosis-inducing effect on HCT116 colon cancer cells^12,14^. Sulforaphane and its metabolites can also induce cytotoxicity in human colon adenocarcinoma HT29 cells^16^ and apoptotic cell death in LNCaP prostate cancer cells^17^. Additionally, the microgreens from Thai rat-tailed radish have been reported to be a source of nutrients and antioxidants as they contain proteins, fibers, ascorbic acid, and β-carotene together with the antioxidant activity^18^.

New knowledge of extracellular vesicles (EVs) is emerging. EVs are nano-sized vesicles comprising complex biochemicals (viz., proteins, lipids, and nucleic acids) and small metabolites^19–21^ that can be released from any cells (human or animal), including plants^21–29^. EVs can be classified into exomeres, exosomes, ectosomes, migrasomes, apoptotic bodies, and oncosomes^30^. Despite various terms used for EVs in previous reports, EVs was the term used in this article. EVs, albeit released externally, resemble their parent cells, demonstrating high biocompatibility, and low immunogenicity, and they can deliver signals to target cells. EVs have also been demonstrated to be stable delivery systems under simulated stomach^31,32^ and intestine conditions^21^. These properties indicate their potential for therapeutics (i.e., cancer treatment^33^, pathogen vaccination^34^, immunomodulation^35^, regenerative therapy^36^), and drug delivery systems^29,37^.

Plant-derived EVs exert various bioactivities (e.g., induction of transporter protein expression^27^, inhibition of mice colitis^26^, protection of mice liver damage^38^, and suppression of cancer proliferation^25^). Plant-derived EVs show potential for cancer treatment based on their absence of toxicity, and easy internalization by mammalian cells^39^. Plant-derived EVs have been reported to deliver various types of therapeutic agents (e.g., anticancer drugs, small interfering RNAs (siRNAs), microRNAs (miRNAs)), or poorly soluble natural compounds (e.g., curcumin)^39^. Plant-derived EVs have been studied widely from fruits, vegetables, and spices^19^. As a drug delivery system, grapefruit-^29^ and ginger-derived EVs^40^ delivered the chemotherapeutic agents to the tumor sites.

As anticancer agents, lemon-derived EVs exerted antiproliferative activity and apoptosis-inducing activities in lung cancer, colon cancer, and leukemia cell lines^25^. EVs reduced the growth of human colorectal adenocarcinoma by down-regulating acetyl-CoA carboxylase alpha^41^, which relates to the synthesis of cancer cell fatty acid that cancer cells employ for energy storage, membrane proliferation, and the generation of signaling molecules^42^. Intraperitoneal injection of ginseng-derived EVs was effective against melanoma after intraperitoneal injection based on an immunomodulatory effect promoting a T-helper type 1 cell response in melanoma-bearing mouse models^28^. Garlic-derived EVs presented the antiproliferative activity in A498 kidney cancer and A549 colon cancer cell lines by arresting the cells at the S phase, inducing the intrinsic apoptosis pathway, and reducing vascular endothelial growth factor expression, which relates to the cancer cell vasculogenesis^24^.

Colon cancer was ranked third among the most common cancers after prostate and breast cancer^43^. The mortality rate was predicted to increase over the next decade, rising especially among young adults due to shifting lifestyles and Westernization of the diet^43,44^. The risk factors include genetics, sedentary lifestyle, obesity, poor diet, alcohol intake, smoking, chronic inflammatory bowel disease, and gut microbiota^43^. Different therapeutic advancements (e.g., surgery, targeted therapy, radiation therapy, and chemotherapy) have inhibited cancer progression and prolonged overall survival^43^. Due to the different subtypes and phenotypes and their variable responses to specific therapy, more treatment options are needed.

EVs isolated from vegetables were the focus of this study. The only plant in the Braciacaceae family previously used as a source of EVs were the flowering head and stalk juice of broccoli (*Brassica oleracea*)^26^. The prevention of colitis in mice of broccoli-derived EVs was attributed to sulforaphane^26^. The bioactive compounds of the extract from Thai rat-tailed radish that contributed to the anticancer activity in colon cancer cells^12,14,45^ were sulforaphane and sulforaphene^13,45^. The crude extract of Thai rat-tailed radish has been reported for its anticancer action. Since Thai rat-tailed radish has not been previously proposed as a source of EVs, we sought to isolate the EVs from Thai rat-tailed radish. We hypothesized that this plant might be a source of anticancer EVs and have therapeutic potential. The present research, thus, aimed to isolate EVs from the microgreens of Thai rat-tailed radish, then demonstrate using them as a source of EVs and determine the anti-cancer activity in HCT116 adenocarcinoma cells.

EVs can be prepared by different means with varying recoveries and specificities (e.g., size exclusion chromatography, tangential flow filtration, membrane affinity columns, and differential centrifugation)^46^. Remarkably, a commonly used technique for isolating plant originated-EVs is differential centrifugation^25–27,47–49^. We used differential centrifugation and filtration to determine their practicability in any laboratory setting for preparing plant-derived EVs.

Before bioactivity testing, characterization is essential to address whether EVs can be obtained from the proposed method and plant source. In order to investigate the properties of EVs from Thai rat-tailed radish microgreens, we determined the size distribution (qualitative representation) and observed the morphologies under the electron microscope in transmission and scanning transmission modes. Dynamic light scattering was employed to confirm the size distribution from the transmission electron microscope and determine the zeta potential (stability representation). The biological macromolecular compositions were studied using FTIR analysis^50–54^, while the chemical micromolecular compositions were determined using HPLC analysis. The resultant data indicate that the differential centrifugation technique is capable of isolating EVs from plants. Likewise, EVs from Thai rat-tailed radish microgreens possess a nanovesicular structure comprising natural macromolecules as the major components, specifically a protein with a β-pleated sheet for a secondary structure. EVs, moreover, showed the anti-cancer activity against HCT116 colon cancer cells in improved selectivity and safety superior to microgreen extract.

## Results

### Extraction yield of *Raphanus sativus* L. var. *caudatus* Alef microgreens

**Figure 1:**
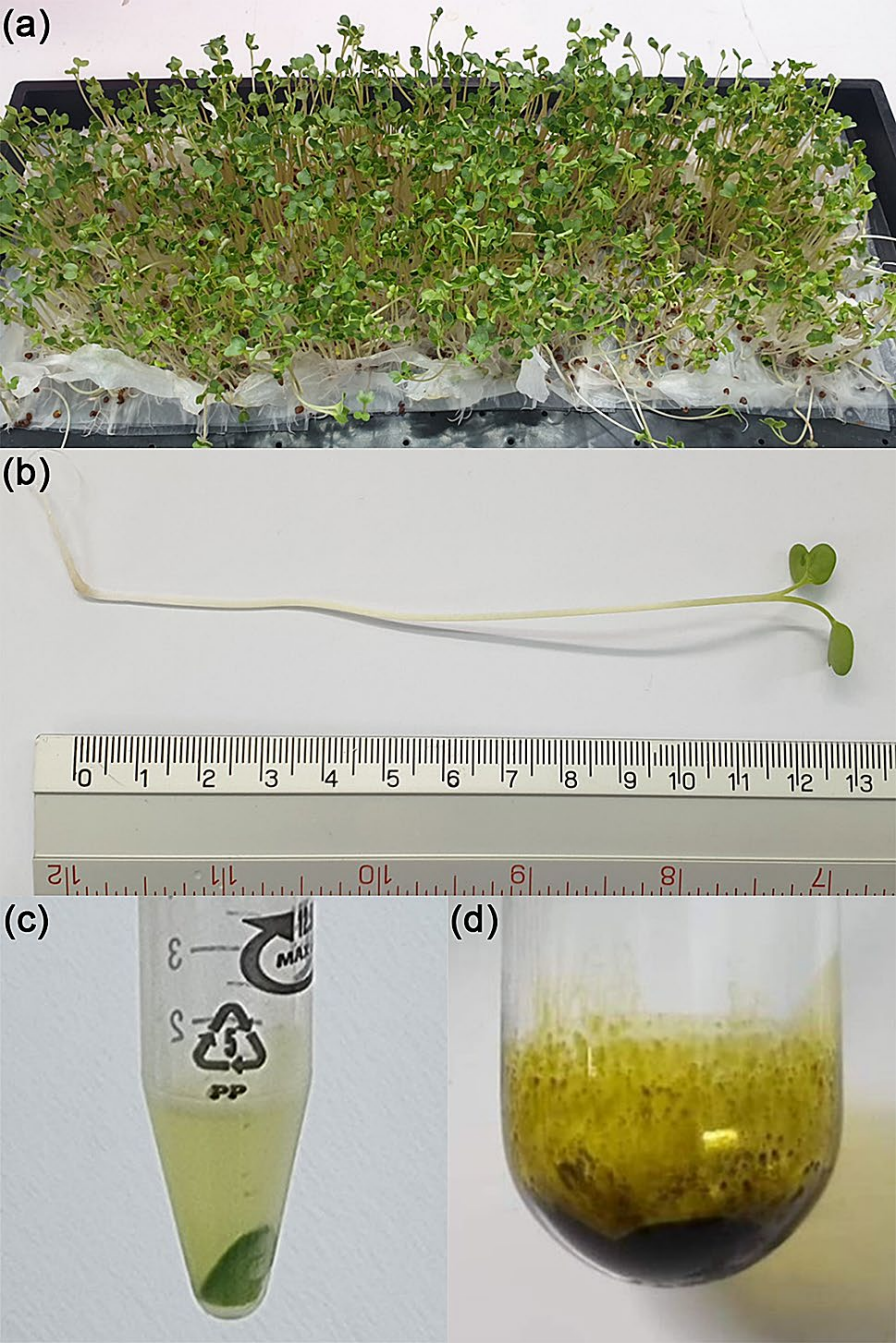
*Raphanus sativus* L. var. *caudatus* Alef (Thai rat-tailed radish) microgreens. (**a**) Cultivated seedlings from 20 g seeds grown for 6–7 days on paper towels. (**b**) Harvested microgreens 10–13 cm in length. (**c**) EVs in the supernatant part at the final step after differential centrifugation; and (**d**) DCM crude extract of microgreens (0.16% yield per fresh weight).

The microgreens of *Raphanus sativus* L. var. *caudatus* Alef (Fig. 1a) (99.21 ± 10.42 g) were grown from the dry seed (20 g) and harvested on days 6–7, when their length reached 10–13 cm (Fig. 1b). This microgreen was used to prepare extracellular vesicle (EVs) by differential centrifugation (Fig. 1c) and was extracted by liquid phase extraction. Dichloromethane (DCM) represented the nonpolar phase, previously used to extract the isothiocyanates (ITCs)^11–14^. After the DCM layer was collected, the solvent was removed by a rotary evaporator, yielding a dry crude DCM extract. The obtained yield of the DCM crude extract was 0.16% w/w per fresh weight (Fig. 1d).

### Physical properties of EVs

**Figure 2:**
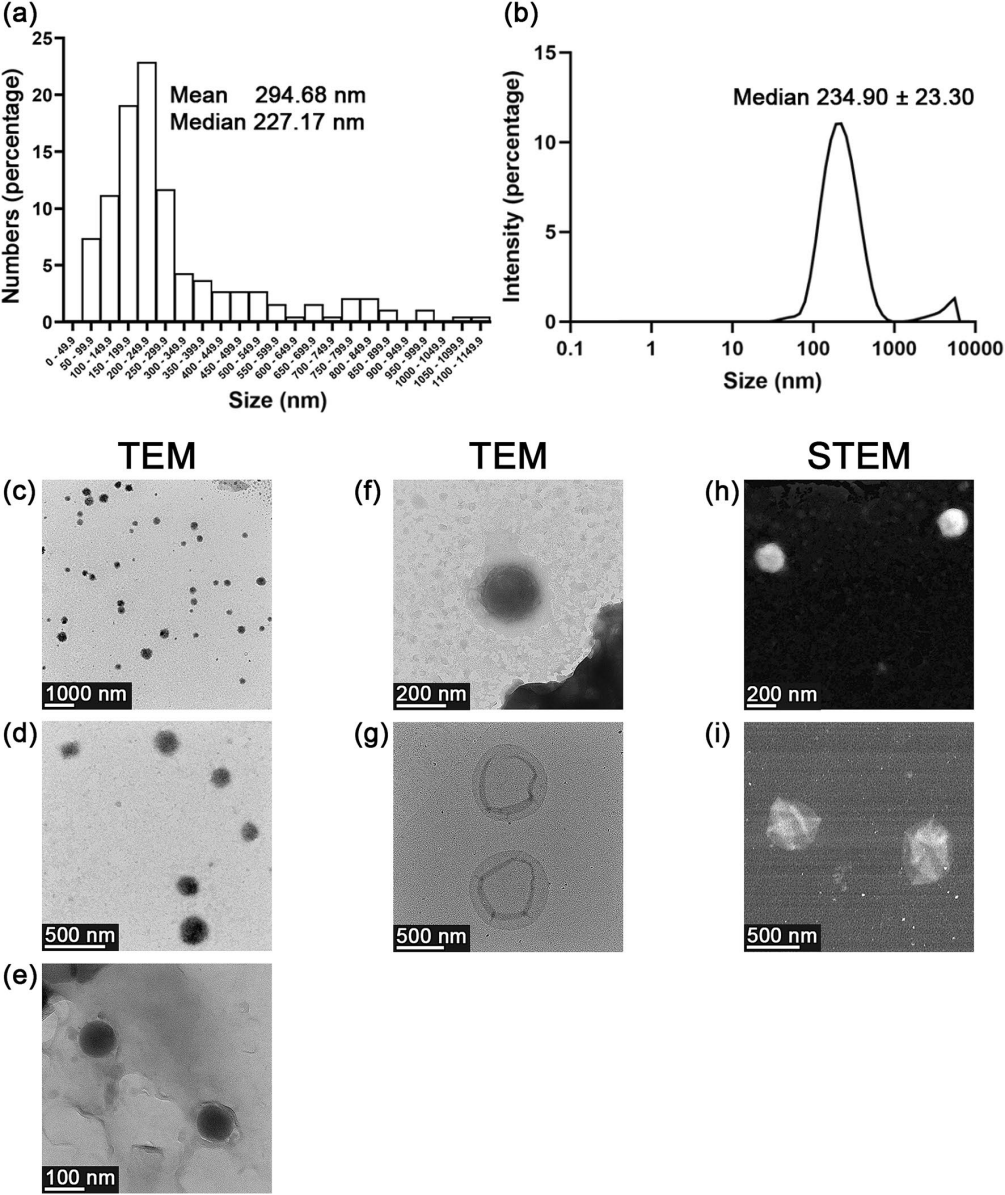
Physical properties of EVs derived *from Raphanus sativus* L. var. *caudatus* Alef microgreens. Particle size distribution as measured by (**a**) transmission electron microscopy (TEM) and (**b**) dynamic light scattering (DLS). Images from TEM showing the wide-field and close-up appearance of EVs at (**c**) 5500x, (**d**) 22,500x, and (**e**) 74,000 × magnifications, respectively. Images from TEM of (**f**) spherical EVs at 46,000 × magnification and (**g**) cup-shaped EVs at 17,500 × magnification. Images from scanning transmission electron microscope (STEM) of (**h**) spherical EVs at 64,200 × magnification (**i**) and cup-shaped EVs at 45,400 × magnification.

EVs derived from the microgreens of *Raphanus sativus* L. var. *caudatus* Alef were isolated by differential centrifugation. The size distribution of EVs was determined using a transmission electron microscope (TEM). EVs (a total of 188 vesicles) were randomly captured from 17 fields and shown in various sizes. The size distribution determined by the frequency of each size range was plotted as a bar graph (Fig. 2a). The respective mean and median values of EVs size were 294.68 and 227.17 nm. The size distribution of EVs was confirmed by dynamic light scattering (DLS) (Fig. 2b). The prominent distinctive peak (95.20 ± 4.14% population) of EVs demonstrated a particle size ranging between 30 and 1000 nm with a median of 234.90 ± 23.30 nm and polydispersity index (PDI) of 0.293 ± 0.019 (Fig. 2b). Results from TEM and DLS confirmed that the small-sized EVs were obtained under the studied conditions and shown in the similar size range. Images from TEM were captured in the different magnifications to demonstrate the wide-field and close-up appearances of EVs as recommended by the minimal information for studies of extracellular vesicles (MISEV) 2018 guidelines^46^ (Fig. 2c, d, and e). The images obtained from TEM and scanning transmission electron microscope (STEM) reveal the nano-size of the EVs (between 200 and 1000 nm) in conformity to DLS. In the present study, EVs had various appearances based on TEM/STEM, including spherical (Fig. 2f and h) and cup-shaped (Fig. 2g and i). To the best of our observation, no protein aggregates were seen under the electron microscope. The zeta potential of the EVs, as measured by DLS, was –11.68 ± 1.77 mV.

### Biological compositions of EVs

**Figure 3:**
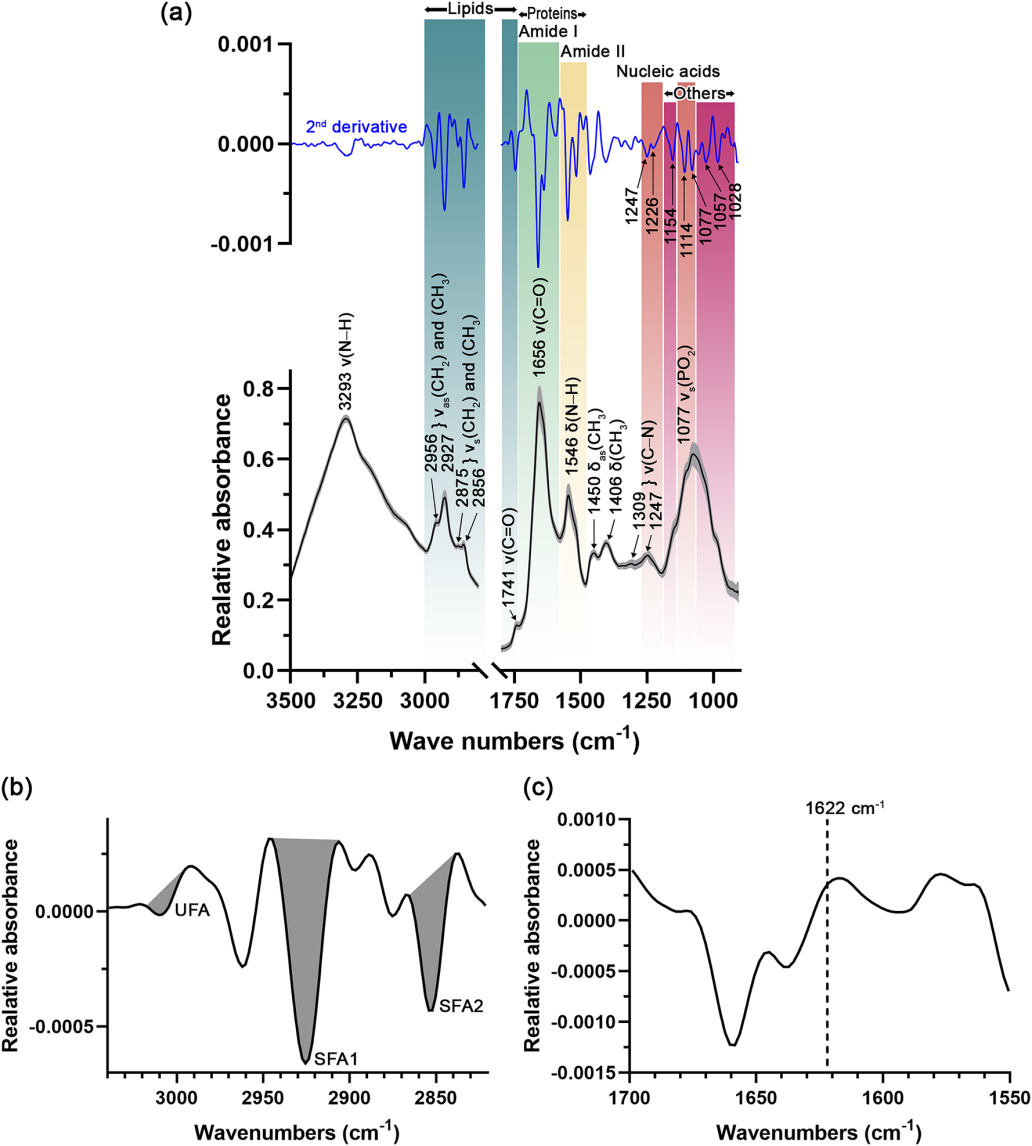
(**a**) Primary (black line) and second derivative (blue line) FTIR spectra of biological compositions of EVs derived from *Raphanus sativus* L. var. *caudatus* Alef microgreens after background (PBS) subtraction, Savitzky-Golay smoothing (3rd-degree polynomial with 13 smoothing points), and Extended Multiplicative Signal Correction (EMSC) normalization. An averaged spectrum from 118 spectra was presented in black and the standard deviation was indicated by the gray parallel bands. ν = stretching vibration, ν_as_ = asymmetrical stretching vibration, ν_s_ = symmetrical stretching vibration, and δ = bending vibration. (**b**) Second derivative spectrum of EVs in lipid region. UFA = unsaturated fatty acid region (3017–2996 cm^–1^), SFA1 = saturated fatty acid region 1 (2944–2906 cm^–1^), and SFA2 = saturated fatty acid region 2 (2865–2839 cm^–1^). Gray areas indicate the area integration range. (**c**) Second derivative spectrum of EVs in protein region, presenting the absence of 1622 cm^–1^ band of non-native intermolecular ß-sheet from protein aggregates.

The FTIR technique was performed to ascertain the main biological molecules in EVs. Representative FTIR primary spectra of the EVs are presented in Fig. 3a. The bands of the different functional groups of biocomponents are presented in three main regions: lipids, proteins, and nucleic acids. Different biological molecular composition were classified into (1) lipids (2972–2844 and 1754–1736 cm^–1^), (2) amide I (1674–1627 cm^–1^), (3) amide II (1557–1509 cm^–1^), (4) nucleic acids (1267–1215 and 1124–1066 cm^–1^), and (5) other biological molecular compositions (1169–1146 and 1059–921 cm^–1^). The assigned bands for the specific biomolecules are summarized in Table S1 (Supplementary information). A strong peak at 3293 cm^–1^ was designated as the N–H stretching vibration of the peptide groups and termed amide A. In the lipid region (3000–2800 cm^–1^), the respective peaks at 2956 and 2927 cm^–1^ were designated the asymmetrical stretching vibration of the CH_2_ and CH_3_ acyl chain lipids. The peaks at 2875 and 2856 cm^–1^ depict the symmetrical stretching vibration of the CH_2_ and CH_3_ acyl chain lipids. The proteins region (1800–1350 cm^–1^) shows two predominant peaks at 1656 and 1546 cm^–1^ representing carbonyl stretching vibration of the peptide backbone of amide I and N–H bending vibration of peptides of amide II, respectively. A weak peak at 1741 cm^–1^ shows the absorption of the stretching vibration of the ester carbonyl groups (viz., phospholipids, triglycerides, and cholesterols). The peak at 1450 cm^–1^ represents the asymmetrical bending of CH_3_ of the lipid acyl chains, while the peak at 1406 cm^–1^ represents the CH_3_ bending vibration of acyl residues of the lipids and proteins. A nucleic acid region (1267–1215 and 1124–1066 cm^–1^) shows one prominent peak at 1077 cm^–1^ designated the symmetrical stretching vibration of the PO_2_ in the DNA and RNA. The remaining two minor peaks at 1309 and 1247 cm^–1^ demonstrate the C–N stretching of proteins, referring to the α-helical conformation of amide III peak. It is the first report of the FTIR peak characteristics of EVs derived from plant material. The peaks in the present study are similar to those found in the EVs derived from mammals in other studies^50–54^.

The second derivative spectrum of the lipid region was designated as saturated and unsaturated fatty acid regions (Fig. 3b). The peak at 3010 cm^–1^ was assigned as =CH stretching vibration of unsaturated lipids. The respective peaks at 2925 and 2852 cm^–1^ were designated as CH_2_ asymmetrical stretching of saturated fatty acid region 1 (SFA1), and CH_2_ symmetrical stretching vibrations of saturated fatty acid region 2 (SFA2). The differential centrifugation method at low *g*-force might lead to co-isolate non-vesicular materials (e.g., protein aggregates) that could contaminate fractions. However, in our study, no protein aggregates contaminants were detected under electron microscopy (Fig. 2c–i). It was also confirmed, using the second derivative FTIR spectrum, by the absence of the peak assigned for protein aggregates (or non-native intermolecular ß-sheet) at 1622 cm^–1^ in the amide I peak^51,55,56^ (Fig. 3c).

The second derivative spectra (Fig. 3a, blue line) were presented to substantiate the peaks and bands shown in the primary spectra (Fig. 3a, black line) and indicate the assigned peaks of nucleic acids and other biological molecular compositions. In the nucleic acid region, the asymmetrical stretching vibration of PO_2_ moieties was assigned for 1247 cm^–1^ (detected in RNA) and 1226 cm^–1^ (detected in DNA) (Fig. 3a). The different peak assignments for RNA and DNA at the respective 1247 and 1226 cm^–1^ were reported to be due to the different conformation of RNA as A-form and DNA as B-form of nucleic acids^57^. The peak at 1114 cm^–1^ represents asymmetrical stretching vibration of PO_2_ moieties of RNA and stretching vibration of the skeletal structure of the C2′ hydroxyl group of ribose, only found in RNA. Hence, the disappearance of the peak at 1114 cm^–1^ in addition to the characteristic peak of DNA at 1226 cm^–1^ (assigned for asymmetrical stretching vibration of phosphate moieties of DNA) could indicate the presence of DNA. The peak at 1077 cm^–1^ shows a symmetrical stretching vibration of the PO_2_ in the DNA and RNA as was presented in the primary spectra. In other biological molecular composition regions, the peak at 1154 cm^–1^ represents the C–O stretching and C–O–H bending vibration of carbohydrates. The peak at 1057 cm^–1^ depicts the C–O stretching of polysaccharides. The peak at 1,028 cm^–1^ was C–O stretching and C–OH bending vibration of oligosaccharides and polysaccharides.

**Figure 4:**
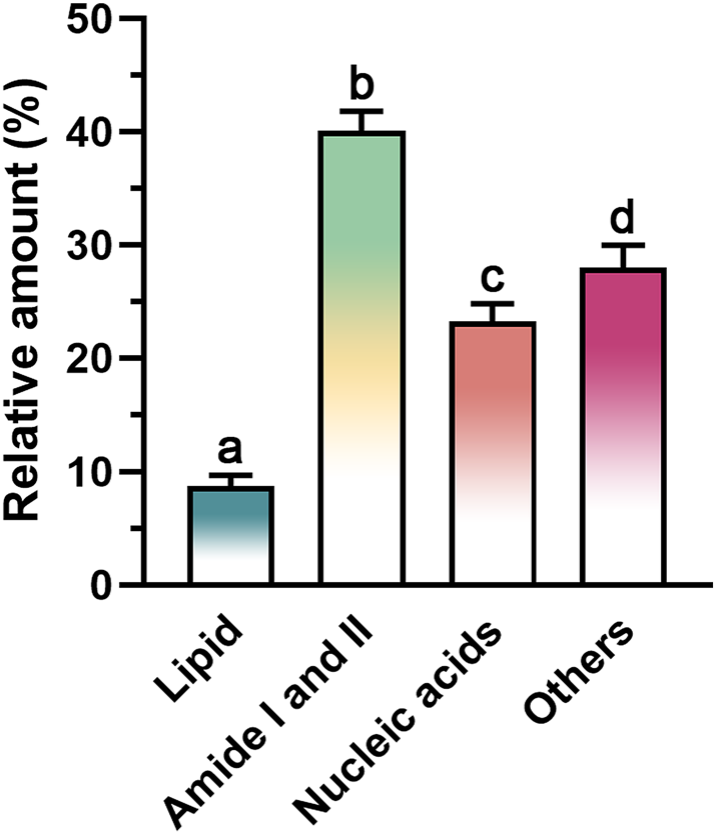
Relative amount of biological compositions of EVs derived from *Raphanus sativus* L. var. *caudatus* Alef microgreens as determined by specific area integration under the second derivative spectra. Different letters indicate a statistical difference between groups (*p*-value < 0.05).

The integrated areas under the second derivative spectra in each specific region in Fig. 3 refer to the relative amounts of individual biological molecular compositions: lipids, proteins (amide I and amide II), nucleic acids, and others (Fig. 4). Proteins represented as amide I and amide II, exerted a significantly higher abundance over the other integrated regions, as shown by the 40.06 ± 1.76% relative amounts followed by other components (27.97 ± 2.01%), nucleic acids (23.24 ± 1.59%), and lipids (8.73 ± 0.97%). Hence, protein is the primary biological composition comprising EVs. The saturated fatty acid and unsaturated fatty acid were also determined, which were 3.74 ± 0.45% and 96.26 ± 0.45%, respectively. The saturated fatty acid-to-unsaturated fatty acid ratio was 26.00 ± 3.46, while the lipid-to-protein ratio was 0.23 ± 0.01.

**Figure 5:**
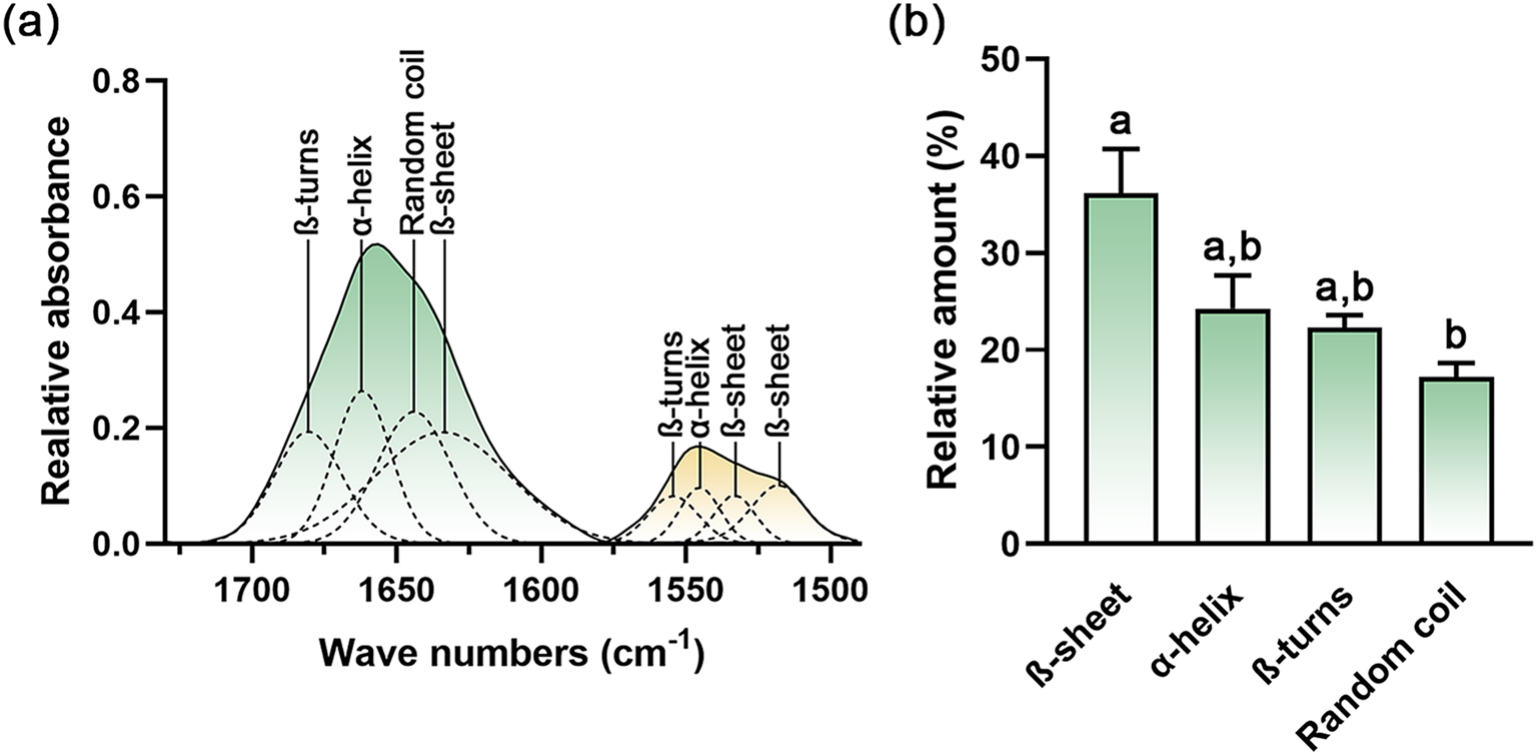
Protein secondary structures containing EVs derived from *Raphanus sativus* L. var. *caudatus* Alef microgreens were determined by (**a**) curve-fitting with the 50% Gaussian and Lorentzian function in the amide I and amide II regions (RMS error 0.002). (**b**) The relative amount of protein secondary structure was determined by specific area integration. Different letters indicate a statistical difference between groups (*p-*value < 0.05).

Curve fitting analyses of the amide I and amide II regions were employed to study the conformation of proteins and specify their secondary structures. In amide I region (1730–1580 cm^–1^), β-turns, α-helix, random coil, and β-sheet are indicated by the peaks at 1676–1680 cm^–1^, 1659–1661 cm^–1^, 1641–1642 cm^–1^, and 1627–1632 cm^–1^, respectively (Fig. 5a). For amide II region (1580–1490 cm^–1^), β-turns, α-helix, and β-sheet are assigned by the peaks at 1554–1558 cm^–1^, 1546–1548 cm^–1^, 1535–1537 cm^–1^, and 1519–1520 cm^–1^, respectively.

The area integration under each specific band was assessed to determine the relative amount of each specific protein secondary structure. After summing up the area of the same secondary structures from different band peak positions, the β-sheet had the greatest abundance (viz., 36.16 ± 4.58%) (Fig. 5b). The α-helix, β-turns, and random coil are presented by 24.27 ± 3.39%, 22.32 ± 1.27%, and 17.25 ± 1.39%, respectively.

**Figure 6:**
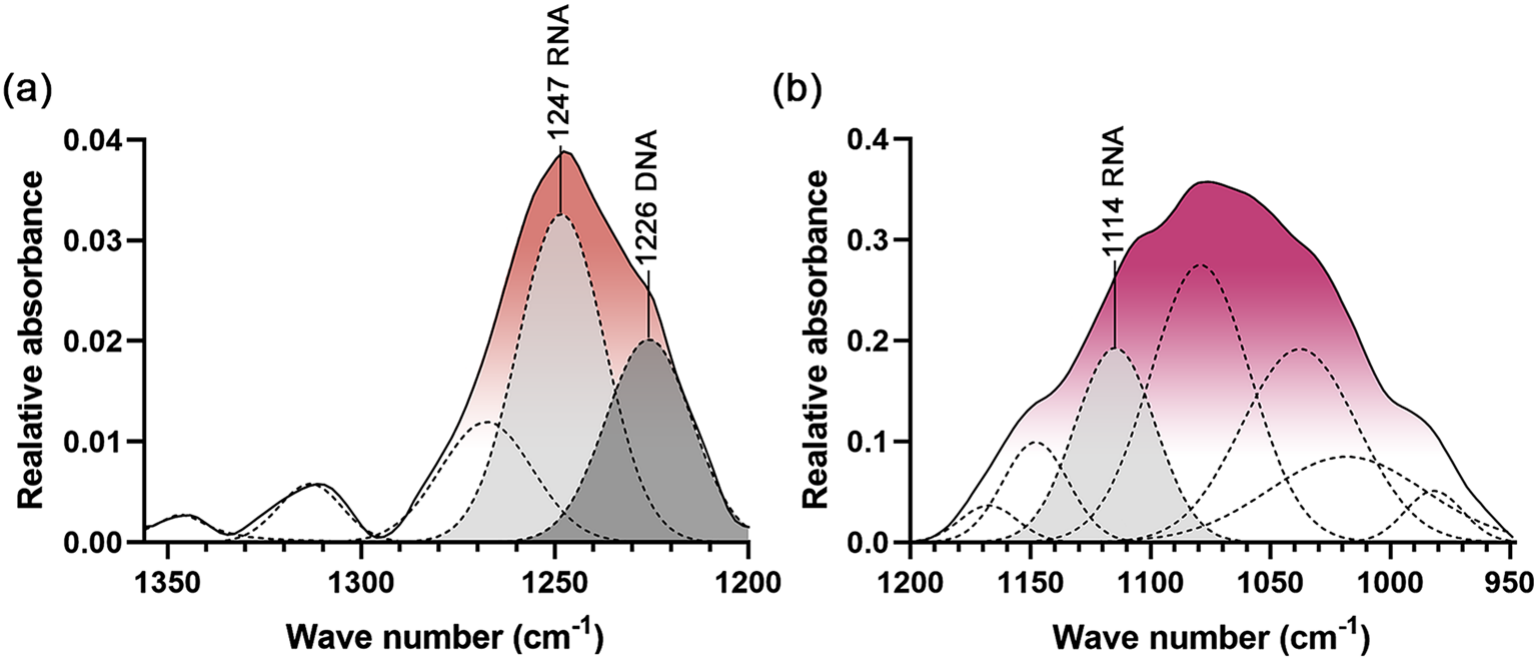
Designated peaks of DNA and RNA containing in EVs derived from *Raphanus sativus* L. var. *caudatus* Alef microgreens determined by curve-fitting with the 50% Gaussian and Lorentzian function in (**a**) 1356–1200 region (RMS error 0.001) and (**b**) 1,200–950 region (RMS error 0.002).

To determine the existence of DNA and RNA in EVs, curve fitting analyses of the nucleic acid and other biocomponent regions were performed. In the regions 1356–1200 and 1200–950 cm^–1^, DNA is indicated by the peak at 1226 cm^–1^ of asymmetric stretching of phosphate moieties (Fig. 6a). RNA is indicated by the peaks at 1247 and 1114 of asymmetric stretching of phosphate and stretching vibration of skeletal structure around hydroxyl group at C2′ of ribose (Fig. 6a and b).

NanoDrop analysis was also performed to determine the amount of DNA and RNA in EVs. DNA and RNA contents in 1000 µg/ml EVs were 106.33 ± 5.51 and 86.40 ± 0.80 µg/ml, respectively. As absorbance ratios of 260 nm/280 nm are used to determine the DNA and RNA purity, if the ratio is appreciably lower in either case (less than 1.8 and 2, respectively), it may indicate the existence of proteins in the EVs. The respective 260/280 ratio of EVs in DNA and RNA determination were 1.47 ± 0.00306 and 1.47 ± 0.0135 thus indicating existing of protein in the EVs sample.

### Chemical compositions of EVs

**Figure 7:**
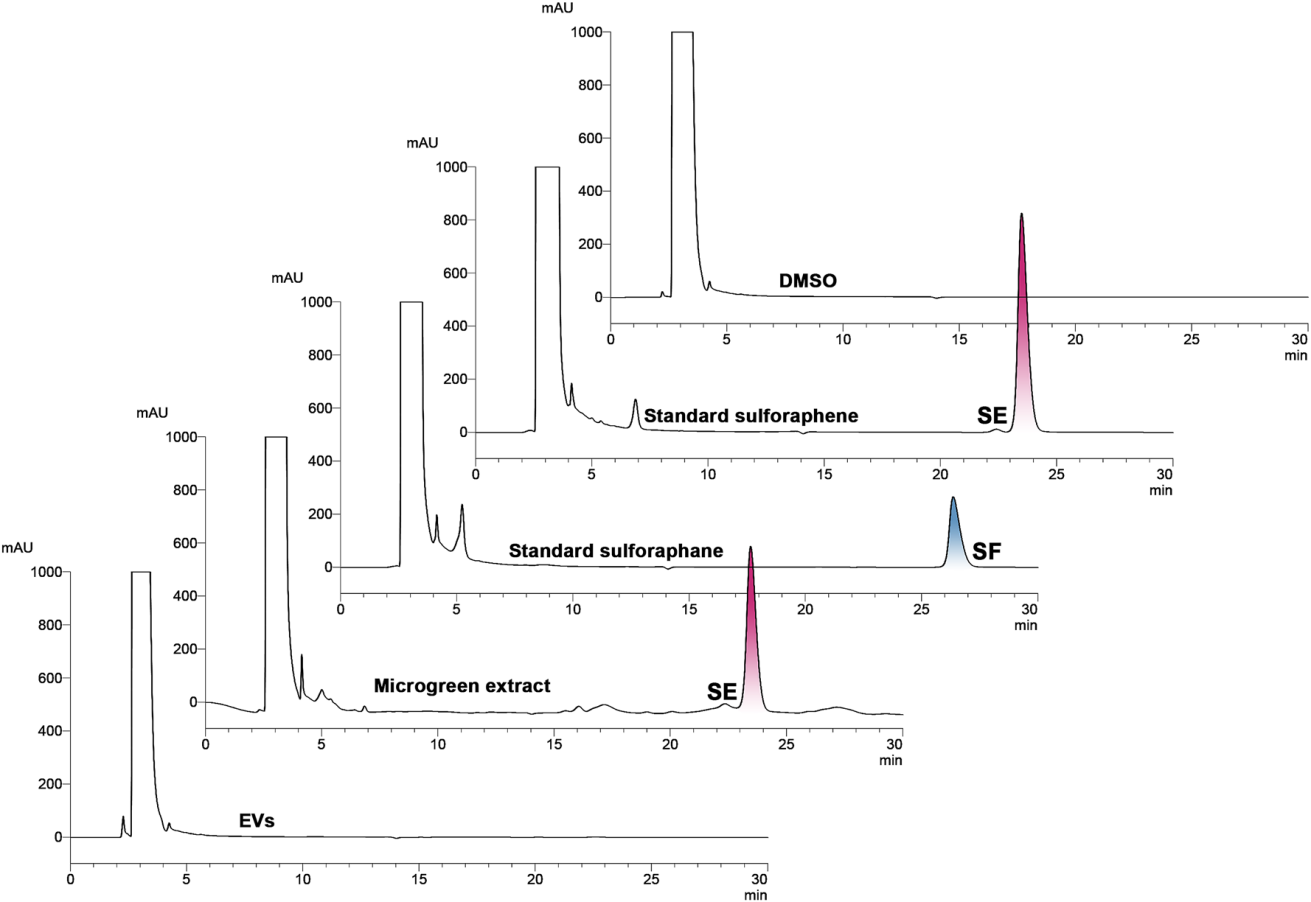
HPLC chromatograms of DMSO, standard sulforaphene (1 mg/ml), sulforaphane (1 mg/ml), microgreen extract (20 mg/ml), and EVs (6.67 mg/ml).

Sulforaphene (SE) and sulforaphane (SF) are isothiocyanates (ITCs), and they are chemical biomarkers found in the Brassicaceae plants (including Thai rat-tailed radish)^11–14,58,59^. These two compounds were identified in the EVs and in the microgreen extract by comparing the peak retention time with the standard SF and SE compounds. The respective retention time of standard sulforaphene and sulforaphane was 23.50 ± 0.005 and 26.37 ± 0.001 min (Table S2, Supplementary information). The ITC content was determined in the DCM crude extract of the microgreens and the EVs base on the peak height in the HPLC chromatograms detected at 210 nm (Fig. 7). The DCM crude extract from the microgreens showed only SE in the amount of 0.061 ± 0.002 mg/g fresh weight at the retention time 23.47 ± 0.006 min. By comparison, neither SE nor SF was detected in the EVs, suggesting a very tiny amount or none in the sample according to the limit of detection (SE 0.34 and SF 0.36 µg/ml) and limit of quantification (SE 1.02 and SF 1.08 µg/ml) of analyzing condition^59^.

### Antiproliferative effect of EVs

**Figure 8:**
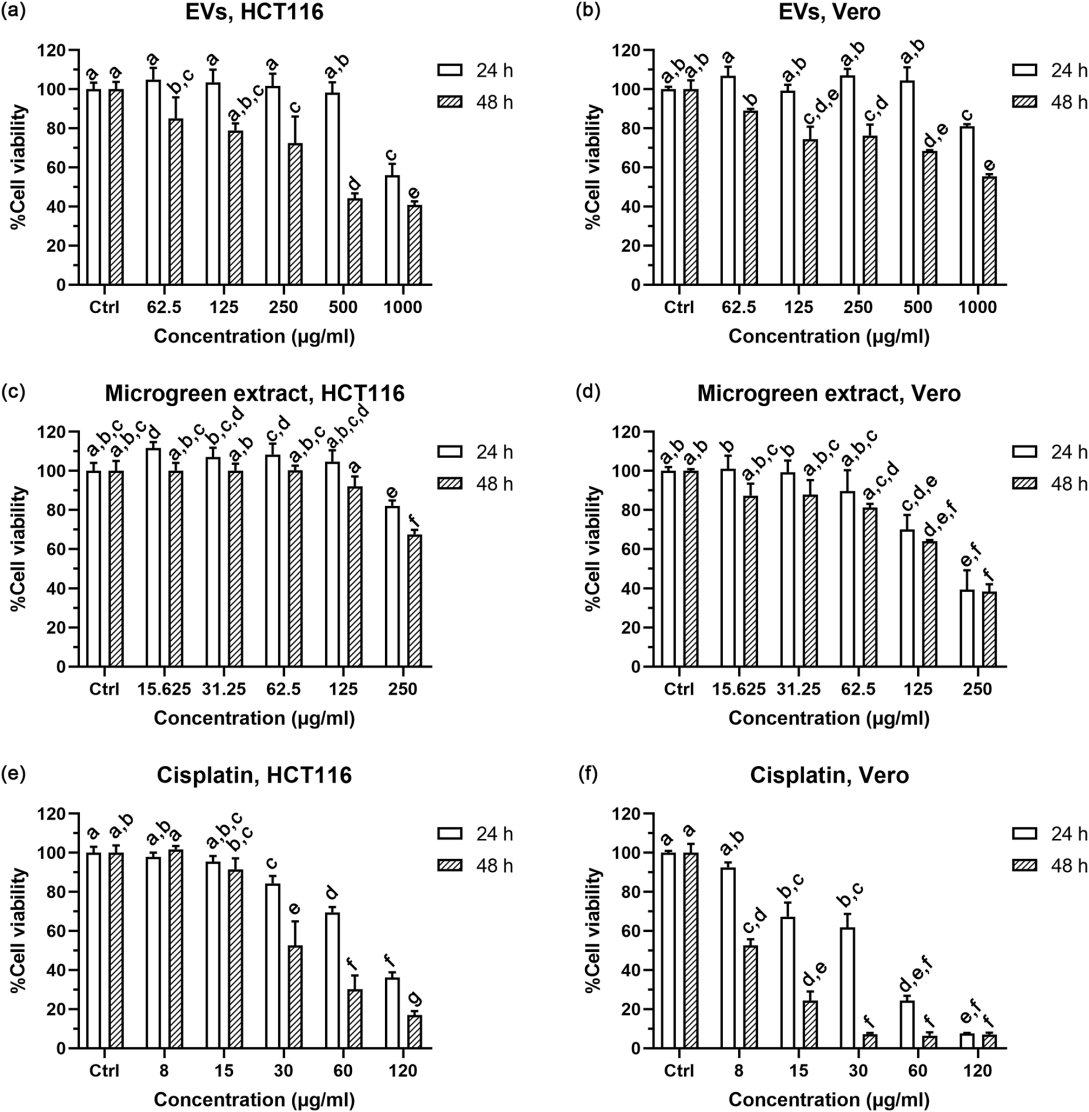
The antiproliferative effect after 24 and 48 h treatment by neutral red uptake assay of (**a**) EVs in HCT116 cells, and (**b**) Vero cells; (**c**) microgreen extract in HCT116 cells and (**d**) Vero cells; (**e**) Cisplatin—a positive control—in HCT116 cells, and (**f**) Vero cells. Different letters indicate a statistical difference in the sample at different concentrations and exposure times in the same cell line (*p-*value < 0.05).

The biological activity of EVs was investigated by determining the antiproliferative effect despite not detecting any bioactive ITCs in the EVs. In order to screen the anti-cancer activity of EVs against colorectal cancer, the HCT116 cell line was used as the cancer cell model. Vero cells—representative of normal cells—were used to determine the selectivity of the antiproliferative effect between cancer cells and non-cancer cells. In the event that the %cell viability was higher than 50% at the maximum concentration, the IC_50_ would be reported at this maximum concentration. The neutral red uptake assay demonstrated the antiproliferative effect of EVs against HCT116 colon cancer cells in a concentration- and time-dependent manner (Fig. 8a) with a 50% inhibitory concentration (IC_50_) of > 1000 and 451.02 µg/ml at 24 and 48 h incubation time, respectively. EVs showed lower antiproliferation against Vero cells with an IC_50_ > 1000 µg/ml at both exposure times (Fig. 8b). EVs exhibited significantly higher antiproliferation at 48 h than at 24 h (*p* < 0.05) at concentrations higher than 62.5 µg/ml in both HCT116 and Vero cells. At 48 h, the selectivity index (SI) of EVs in HCT116 over against Vero cells was > 2.2. Conversely, DCM crude extract from microgreens showed more antiproliferative effect against normal cells (Fig. 8d). Compared to EVs, microgreen extract possessed less antiproliferation in HCT116 cells but greater antiproliferation in normal cells (Fig. 8c,d). The IC_50_ of microgreen extract was > 250 µg/ml in HCT116 at both exposure times; and equal to 207.82 µg/ml and 195.89 µg/ml in Vero cells at 24 and 48 h, respectively (Fig. 8c,d). Cisplatin, a chemotherapeutic drug used as a positive control, exhibited stronger cytotoxicity in both HCT116 and Vero cells at both incubation times (Fig. 8e,f) than the microgreen extract and EVs. Higher cytotoxicity of cisplatin was observed in the Vero cell cells (IC_50_ = 36.20 and 5.88 µg/ml at 24 and 48 h, respectively) than that of the HCT116 cell line (IC_50_ = 94.84 and 24.69 µg/ml at 24 and 48 h, respectively).

A higher antiproliferative effect of the microgreen extract than EVs might be due to the presence of sulforaphene in the DCM extract, which was the absence in the EVs confirmed by the HPLC analysis. Hence sulforaphene was not attributed to the antiproliferative activity of EVs. Our previous studies demonstrated that the DCM extracts from the pod, flower, and dry seed of Thai rat-tailed radish induced HCT116 colon cancer cell death via apoptosis death mode as contributed by the isothiocyanates—sulforaphane and sulforaphane^12,13^. The selectivity of the extract from the edible part of Thai rat-tailed radish toward the colon HCT116 cells after 3 weeks of planting was 2.2 and increased with age (7 weeks, SI = 6.5)^13^. Interestingly, the respective selectivity index (SI) ranked from high to low vis-à-vis the colon cancer cells at 48 h was EVs (> 2.2), microgreen extract (< 0.8), and cisplatin (0.2). The selectivity rank in the colon cancer cell line at 24 h had the same order. EVs isolated from the microgreens of Thai rat-tailed radish did not contain micromolecules—ITCs—as found in the DCM crude extract but rather macromolecules such as proteins, lipids, and nucleic acids. The presence of the macromolecules in EVs may contribute to its antiproliferation effect. These results assured that EVs have the potential to be tested or used at a higher level of study according to more safety profiles.

## Discussion

The plant is the natural source of various beneficial nutrients and pharmaceutically active compounds. Using conventional organic solvent extraction and a unique extraction protocol, crude extracts can acquire some specific groups of compounds^60^. In this study, the microgreens were ground and mixed with DI water. During this step, plant cells were damaged, and the myrosinase enzyme was released and exposed to polar secondary plant metabolites—glucosinolates—abundant in Brassicaceae vegetables. Glucosinolates underwent enzymatic reaction and converted into more nonpolar and biologically active isothiocyanates. These isothiocyanates were then readily and simultaneously partitioned into the DCM layer. Along with the specific chemical class of compounds, certain biological molecules are eliminated by the organic solvent (i.e., protein denaturation)^61^ and alteration of nucleic acid structures and activity by affecting the water activity and dielectric constant^62^.

Moreover, a relatively large amount of plant material and an organic solvent is needed to extract to yield a tiny portion of crude extracts or pure chemical compounds^63,64^. Furthermore, due to the low yield, large quantities might have to be consumed to exert any clinical effect. These are not uncommon limitations when developing plant-derived extracts or compounds for clinical use. Thus, consuming the original form of edible plants or vegetables is how a wide range of health-beneficial molecules, including chemical and biological molecules, are made available to humankind. Plants have been indisputable in their value as a source of molecules with therapeutic potential. Hence, the discovery of new pharmacologically-active plant-derived compounds is a challenging task. In this study, we report the technique used to prepare the sample derived from plants using a non-organic solvent generating the extracellular vesicles or EVs.

It is known that edible plant-derived EVs can exert several bioactivities from their biomolecules. In the present study, we prepared the EVs from the microgreens of *Raphanus sativus* L. var. *caudatus* Alef. The EVs derived from the microgreens had a size in the nano range (mean from TEM = 294.68 nm, median from TEM = 227.17 nm, and median from DLS = 234.90 ± 23.30 nm) with a low PDI. The PDI represents the quality of the EVs according to size distribution. A PDI < 0.7 suggests that the samples are suitable to be analyzed by DLS and a PDI ≤ 0.3 represents the homogeneity of the population. A PDI close to zero indicates the monodisperse system of the particle size^65^. Our result had a PDI in the range of 0.2–0.5, as was also reported for other EV types^66^, suggesting size distribution conformity.

Six subtypes of EVs are classified based on release mechanisms and sizes^30^: (a) exomeres represent non-membranous nanoparticles with a size less than 50 nm; (b) exosomes are formed by inward budding and generated by exocytosis with a size range of 30–150 nm; (c) ectosomes or microvesicles are mid-sized EVs (100–1000 nm) released directly from plasma cells via outward budding; (d) migrasomes are released from the tip of retraction fibers after metastasis having large vesicles (500–3000 nm), encapsulating numerous smaller vesicles ranging between 50 and 100 nm and their function needs elucidation; (e) apoptotic bodies (1000–5000 nm) are irregularly shaped, nucleic acid-containing cell fragments released during the terminal stage of apoptosis^67^; and, (f) oncosomes are large-sized EVs (1000–10,000 nm) released from large protrusions of the plasma membrane during amoeboid migration of metastatic prostate cancer cells^30,68^. EVs from microgreens in this study were relatively small-sized compared to the overall EVs, similar to other plant-derived EVs (≤ 1000 nm)^25,27,29,38,69^. The EVs were spherical and cup-shaped; the latter may be due to dehydration during sample preparation for TEM analysis^70^. The negative value of the zeta potential shows the identity of their natural biological membrane as represented in other reports of plant-derived EVs^38,69,71^.

FTIR was used to study the biological molecular compositions in EVs. In addition to proteins, lipids, and nucleic acids, carbohydrates were also detected in EVs derived from different sources e.g., serum^72^, milk^73^, and cell culture-conditioned media^50^. Carbohydrates were reported to be related to the EVs biogenesis^74^, cellular recognition, and uptake of EVs by recipient cells^75^. Carbohydrates were assigned at the same region as nucleic acid (1200–1000 cm^–1^) from the C–O absorption^73^. The lipid-to-protein ratio and the saturated-to-unsaturated fatty acid ratio were previously studied by FTIR spectroscopy and reported. The values varied depending on the size of the EVs. Lipid-to-protein ratios were relatively low (less than 30) in small-sized EVs (30–200 nm) while high ratio values (more than 30) were reported in large-sized EVs (50–600 nm)^76^. The saturated-to-unsaturated fatty acid ratio was also relatively low (ratio of 0.1–0.2) in small EVs compared to large EVs (ratio of 0.3–0.4)^76^. The EVs from this study showed a relatively low lipid-to-protein ratio and low saturated-to-unsaturated fatty acid ratio.

The relatively small size of plant-derived EVs (< 1000 nm)^25,27,29,38,69^ allow them to be delivered via different routes of administration. The administrative routes of EVs are designed to reach the desired in vivo target sites, including intravenous, subcutaneous, intraperitoneal, oral, and intranasal routes^77^. Thus, there is a possibility of these research findings having implications for clinical practice. Many plant-derived EVs have made their way to clinical trials, either as active drug components or drug delivery systems^37^. EVs can be engineered to deliver therapeutic molecules by (1) exogenous loading or directly depositing therapeutic molecules into purified EVs during post-isolation^78^; (2) endogenous loading during EVs biogenesis through the transfection of the parental cells before EVs isolation; and (3) in vivo production of loaded EVs through implantation of genetically engineered cells or in vivo^79^. The following are examples of cases of plant-derived EVs under clinical trial. Grape-juice-derived nanovesicles—taken orally as a dietary supplement—underwent a phase 1 clinical trial (NCT01668849) to evaluate their ability to prevent radiation and chemotherapy-induced oral mucositis during treatment for head and neck tumors^37^. The plant-derived EVs as a delivery systems were loaded with curcumin registered for phase I clinical trial (NCT01294072) to deliver curcumin to normal colon tissue and colon tumor cells in patients undergoing surgery for newly diagnosed colon cancer^23^.

Our group and others reported on the bioactive chemical constituents and bioactivity of *Raphanus sativus* L. var. *caudatus* Alef (Thai rat-tailed radish), particularly its anti-cancer activity. The DCM extracts from Thai rat-tailed radish pods consist of different compounds, including isothiocyanates, glucosinolates, thiocyanates, oxazolidines, indoles alkaloids, flavonoids, dialkyl disulfides, and phenolics^11,12,14,15^. The DCM from the pods demonstrated antioxidant activity and a whitening effect through tyrosinase inhibitory activity^9,11^. In addition, the DCM extract from different parts of Thai rat-tailed radish contained bioactive sulforaphane and sulforaphane, capable of inducing apoptotic cell death in an HCT116 colon cancer cell line^12,13^.

Brassica vegetables are known to contain high levels of vitamins, minerals, dietary fiber, phenolic compounds, and unique compounds, glucosinolates. Sprouts and microgreens of Brassica vegetables have gained acceptance in the population with health concerns because of their health-promoting secondary metabolites and higher nutritional value than those in their mature-leaf counterparts^80–82^. One study reported nutritional contents per 100 g of the edible portion of microgreens from *R. sativus* var. *caudatus*. It contained: protein 6.83 g; fiber 3.70 g; vitamin C 28 mg; β-carotene 569 µg; and chlorophyll 44.5 mg fresh weight^18^. Since microgreens were prepared from Thai rat-tailed radish, the Brassica vegetable, microgreens have been proposed as an excellent source of nutrients and bioactive compounds, which might otherwise be unavailable from mature plants due to unpredictable growing seasons and/or climate change.

Previous work has only focused on the bioactive micromolecules extracted from mature plant parts. Herein, we presented the discovery of EVs derived from the microgreens and on the macromolecules comprising EVs. The proteins, particularly the secondary β-pleated sheet, were a significant component in the EVs derived from microgreens based on FTIR microspectroscopy. Furthermore, the macromolecules of the protein found in the EVs delineated a different composition than that of the micromolecules—sulforaphene—contained in the conventional DCM extracts of the microgreens, detected by HPLC analysis.

In general, the component in the plant-derived EVs can be classified into macromolecules and micromolecules^18,83^. Their bioactivities were reported to come from either macromolecules or micromolecules. For instance, the apple-derived EVs—primarily containing macromolecules of unknown composition—could reduce the toxicity of the chemotherapeutic drug by inhibiting the expression of the OATP 2B1 transporter proteins and decreasing the gastrointestinal tract accumulation^27^. The anticancer activity of lemon-derived EVs can be attributed to the macromolecules (proteins)^25^. In contrast, the respective bioactivity of broccoli^26^ and ginger-derived EVs^21^ was from the micromolecules sulforaphane and shogaol. These two EVs inhibited the dextran sodium sulfate-induced colitis in mice. Additionally, ginseng-derived EVs potentiated the polarization of M2 to M1 macrophages and inhibited melanoma growth that was attributed to their macromolecules (proteins, lipids, and nucleic acids) and active small molecules (ginsenoside Rg3)^28^. Our study shows that the EVs contained all three macromolecules (proteins, lipids, and nucleic acids), with protein being the major component. The micromolecules (sulforaphane and sulforaphene) were not detected in the EVs. EVs, therefore, exhibited the anti-cancer activity against HCT116 adenocarcinoma cells via the major bioactive macromolecules. Nevertheless, higher selectivity index than microgreen DCM extract was found in EVs suggesting the possibility to be studied in a higher level with better safety profile than conventional organic solvent extract.

## Conclusion

Using the simple differential centrifugation method, nano-sized plant-derived EVs can be prepared from the microgreens of *Raphanus sativus* L. var. *caudatus* Alef, as confirmed by the physical properties from DLS and electron microscopy. FTIR and HPLC analysis revealed that protein macromolecules inside the EVs were predominant rather than micromolecules. The characteristic conformation of proteins was a β-pleated sheet secondary structure. Anti-cancer activity in HCT116 colon cancer in a concentration- and time-dependent manner was associated with the EV-containing macromolecules. The outcomes highlight the health benefits of EVs from this plant, and their role in cancer treatment. The knowledge obtained from this study could be useful for developing new anticancer therapeutics for colon cancer.

Notwithstanding our findings, further research is needed on the mechanisms and their activity. The further research step should be a study on the mechanism of action in detail, pharmacology, and in vivo study to close the gap between in vitro research and clinical use. In addition, advancements in chemotherapeutic drug delivery might be made by exogenous loading to achieve more efficacy, but safety and selectivity remain concerns.

## Material and methods

### Material

Phenylmethylsulfonyl fluoride (PMSF) was purchased from AppliChem GmbH (Darmstadt, Germany). Sodium azide was purchased from Avantor (Radnor, PA, USA). D, L-sulforaphane was purchased from Calbiochem (Darmstadt, Germany). L-sulforaphene was purchased from Enzo Life Science (Farmingdale, NY, USA). Dimethyl sulfoxide (DMSO), and Pierce bicinchoninic acid (BCA) protein assay kit were purchased from Fisher Scientific (Loughborough, UK). Hydrochloric acid (HCl), sodium hydroxide (NaOH), and propan-2-ol were purchased from RCI Labscan (Bangkok, Thailand). Chemicals for preparation of buffer solutions (i.e., NaCl, KCl, Na_2_HPO_4_, and KH_2_PO_4_) and neutral red were purchased from Sigma-Aldrich Chemie GmbH (Munich, Germany). Paraformaldehyde, phosphotungstic acid, and anhydrous sodium sulfate were purchased from Sigma-Aldrich Co (St. Louis, MO, USA). Dichloromethane (DCM) and tetrahydrofuran (THF) were purchased from V.S. Chem House (Bangkok, Thailand). Deionized (DI) water was obtained from the PURELAB option-Q (High Wycombe, UK).

### Cell lines and culture

Human colorectal carcinoma HCT116 cells (ATCC CCL-247) and African green monkey kidney Vero cells (ATCC CCL-81) were purchased from the American Type Culture Collection (Manassas, VA, USA). DMEM was used as a culture media supplemented with 10% fetal bovine serum and 1% Pen/Strep (100 unit/ml penicillin and 100 μg/ml streptomycin). The cells were maintained in an incubator at 37 °C with 5% CO_2_ supply.

### Plant material

*Raphanus sativus* L. var. *caudatus* Alef (Thai rat-tailed radish) was a common plant in Thailand and listed in the Botanical Garden Organization (BGO) plant databases (http://www.qsbg.org/database/Botanic_Book%20full%20option/search_detail.asp?botanic_id=2900, accessed on March 10, 2022). Seeds were obtained from a commercially available source in Thailand. Seedlings were grown in Khon Kaen, Thailand (latitude 16.43° and longitude 102.82°) under the natural climate of Thailand for 6–7 days on wet and damp paper towels, which averaged 10–13 cm in length. The experiment was conducted at the Faculty of Pharmaceutical Sciences, Khon Kaen University, Khon Kaen, Thailand during 2019–2021. The aerial part of *Raphanus sativus* L. var. *caudatus* Alef microgreens without roots were used as the source of EVs and microgreen conventional extract. The collection of plant material (KH2562) and the experimental research on microgreens were carried out in accordance with Plant Variety Protection Act B.E. 2542 (1999) Thailand.

### Preparation of EVs

To obtain stable nanosized EVs, EVs were isolated by differential centrifugation. The method was modified following Stanly et al.^84^. Briefly, the microgreens were ground and mixed with PBS containing 0.5 mM PMSF to prevent protein denaturation and 3.3 mM sodium azide to suppress bacterial growth. Next, the slurry was filtered through a cheesecloth. The filtrate was sequentially centrifuged at 1000 × *g*, 4000 × *g*, and 12,000 × *g* for 20 min in each step under 4 °C. The pellet from the last step was resuspended in PBS (1.5 mL) and centrifuged at 4000 × *g* for 20 min to eliminate undesirable co-isolated contaminants; then, the supernatant was collected and filtered through a 0.45 µm syringe filter. The filtrate was collected and used in another experiment within 24 h. The concentration based on protein compositions was determined using Pierce BCA Protein Assay Kit prior to bioactivity testing.

### Electron microscopy

In order to determine the size and clarify morphology, electron microscopy was used to characterize the EVs. As per Frank et al.^85^, the experiment was performed with some modifications. First, EVs were fixed with 4% paraformaldehyde for 30 min and coated on a formvar/carbon supported copper grid 300 mesh. The residual solution was carefully removed from the grid surface using filter paper. Next, EVs were stained using 1% phosphotungstic acid for 30 min. Again, the residual staining solution was carefully removed from the grid surface using filter paper. Finally, the grid was dried overnight at room temperature. TEM images were taken using a Talos F200X scanning/transmission electron microscope (S/TEM) (Thermo Fisher Scientific, Waltham, MA, USA) operating at 200 kV.

Size distribution was studied using TEM analysis by randomly capturing the EVs from 17 fields. The diameter of each EVs was measured using the ImageJ software (version 1.53c, Wayne Rasband, National Institutes of Health, Bethesda, USA). The frequency of each 50-nm diameter range was plotted as a bar graph. The mean and median values of EVs size were determined.

### Dynamic light scattering

In order to confirm the size distribution by TEM analysis and determine the primary stability (represented by zeta potential) of EVs, dynamic light scattering (DLS) was used. EVs were added into DTS1070 folded capillary cells (Malvern Panalytical, UK). The particle size was analyzed using non-invasive backscatter (NIBS) in the manual duration mode. The Zeta potential was analyzed in the automatic mode. The analysis was performed on three different experiments using a Zetasizer Nano ZS (Malvern Panalytical, UK). The data were collected and analyzed by Zetasizer software (version 7.13, Malvern Panalytical, UK).

### FTIR analysis

#### EVs sample preparation

EVs were spotted on the BaF_2_ windows. The droplets were dried on the BaF_2_ windows using a vacuum desiccator for 30 min.

#### Data acquisition

The biocomponents deposited on the Ba_2_F windows were analyzed in the transmission mode of the Bruker Vertex 70v FTIR spectrometer connected to a Bruker Hyperion 2000 FTIR microscope (Bruker optic Inc, Ettlingen, Germany), using synchrotron radiation as the source of IR (Synchrotron Light Research Institute, Thailand). The FTIR spectrometer was employed with a potassium bromide beam splitter and Mercury-Cadmium-Telluride (MCT; HgCdTe) detector cooled with liquid nitrogen. The spectra were scanned from 4000 to 600 cm^–1^ with a 4 cm^–1^ spectral resolution. Forty to fifty spectral analyses were performed on each sample spot with 64 scans/spectrum and a 10 × 10 µm^2^ aperture size. All data were acquired and processed using the OPUS 6.5 software (Bruker optic Inc, Ettlingen, Germany). Water compensation and range selection cut (900 to 3800 cm^–1^) were performed using OPUS 6.5 software. Background (PBS) subtraction, Savitzky-Golay smoothing (3rd-degree polynomial with 13 smoothing points), and Extended Multiplicative Signal Correction (EMSC) normalization were performed using Unscrambler X software (version 10.2, CAMO Software AS, Oslo, Norway). OPUS 6.5 software was also used to obtain the integrated areas from the second derivative spectra, namely: lipids (2972–2844 and 1754–1736 cm^–1^)^86^, amide I (1674–1627 cm^–1^)^50–54^, amide II (1557–1509 cm^–1^)^50–54^, nucleic acids (1267–1215 and 1124–1066 cm^–1^)^87^, and other biological molecular compositions (1169–1146 and 1059–921 cm^–1^)^88,89^. The percent relative amount of integrated area of each biocomponent region was calculated relative to the whole EVs’ region (2972–2844 cm^–1^ and 1754–921 cm^–1^)^90,91^. The lipid–to–protein ratio was calculated from the area integrals of primary spectra in the overall lipid region (2993–2827 cm^–1^ and 1764–1725 cm^–1^) and overall protein region as represented by both the amide I and amide II regions. Then to obtain the saturated–to–unsaturated fatty acid ratio, the second derivative was performed, and the area integral was determined. The regions between 2944 and 2906 and 2865 to 2839 cm^–1^ were calculated to be the saturated fatty acid region; and the regions between 3017 and 2996 cm^–1^ were calculated to be the unsaturated fatty acid. Curve fitting for the amide I and amide II regions was performed to differentiate the secondary protein structures. Curve fitting in the regions of nucleic acid and other biomolecules was performed to demonstrate the existence of DNA and RNA in EVs using the 50% Gaussian and Lorentzian functions. The goodness of fit was assessed using the residual root mean square (RMS) error.

### NanoDrop analysis

The concentration of isolated EVs was determined by a bicinchoninic acid (BCA) assay and diluted to 1,000 µg/ml. DNA and RNA contents were determined using the NanoDrop (BioDrop Duo + Spectrophotometer, Biochrom, Holliston, MA, USA). The 2 µL of EVs was added to the sample compartment. DNA concentration, RNA concentration, and 260/280 ratio were measured.

### Extraction of isothiocyanates (ITCs)

#### Extraction of ITCs from microgreens

To determine the chemical constituents contained in the microgreens, the microgreens was extracted using a conventional organic solvent. The extraction was conducted using the liquid–liquid extraction following the method modified in previous studies^11–14^. Dichloromethane (DCM) represented the nonpolar phase used to extract the isothiocyanates (ITCs). The microgreens were ground with DI water in a 1:1 w/v ratio. The water part was mixed with DCM in a 1:2 v/v ratio and kept for 2 h. The mixture was filtered through cheesecloth. The DCM phase was separated from the water phase using a separatory funnel. The remaining water phase was partitioned with the DCM with the same ratio for another two times. The DCM layer was pooled, collected, and filtered through Whatman™ No. 1. DCM was removed using a rotary evaporator, yielding a dry crude DCM extract.

#### Extraction of ITCs from EVs

In order to compare the chemical micromolecular contents in EVs with a conventional method, ITCs were extracted from EVs. The EVs were identified for the ITCs following the previous lysing method modifications^27^. Briefly, the vesicles were lysed by DCM and centrifuged. Only the DCM layer was collected. The DCM was gently removed by nitrogen purging, yielding a dry sample.

### Identification of ITCs by HPLC

ITCs are the main bioactive component in the plant family Brassicaceae (Cruciferae) and as reported in Thai rat-tailed radish^12–14,59^. The level of ITCs (i.e., sulforaphene and sulforaphane) were determined in both conventional extracts, and EVs derived from the microgreens using HPLC. The HPLC analysis of sulforaphene and sulforaphane was performed as reported^11,13^, using a Prominence-i HPLC (LC–2030C 3D, Shimadzu, Kyoto, Japan) equipped with a diode array detector (Shimadzu, Kyoto, Japan). The stationary phase and guard column comprised the HiQ sil C18W column (4.6 mm × 250 mm, 5 µm) (KYA Technologies Corporation, Tokyo, Japan). The column temperature was set at 25 °C with a flow rate of 1 mL/min. The mobile phase was isocratic elution with 5% tetrahydrofuran (THF) and 95% ultrapure water. The detection wavelength was set at 210, 245, and 254 nm. Labsolution software (version 5.73, Kyoto, Japan) was used to obtain the chromatograms. The DCM dry extract was dissolved in DMSO. The injection volume was 20 µL. Sulforaphene and sulforaphane standard compounds were used to identify the presence of these compounds in the samples by comparing the retention time of the standard peak with the samples. The sulforaphene and sulforaphane contents in the DCM extract and EVs were calculated from the peak height compared to the standard compounds.

### Antiproliferative activity

HCT116 and Vero cells (4 × 10^4^ cells/well) were seeded in a 96-well plate and incubated with varying concentrations of EVs (62.5–1000 µg/ml) and cisplatin (7.5–120 µg/ml) for 24 and 48 h. After the test duration, culture media was replaced by 50 µg/ml neutral red solution and incubated for 2 h. The cells were washed once with PBS and lysed with the acidified propan-2-ol. The absorbance of neutral red was measured at the dual-wavelength of 537 nm and 650 nm by a microplate reader (EnSight multimode plate reader, MA, U.S.). Cisplatin was used as a positive control. The percentage of cell viability was calculated compared to the control (untreated cells) by the Eq. (1). The 50% inhibitory concentration of cell viability (IC_50_) was calculated from curves constructed by plotting cell survival (%) versus samples’ concentration. Selectivity index was calculated by Eq. (2).

1 $$\%\,{\text{Cell viability}} = { }\frac{{\left[ {\left( {{\text{Abs}}_{{537{\text{ control}}}} - {\text{Abs}}_{{660{\text{ control}}}} } \right) - \left( {{\text{Abs}}_{{537{\text{ sample}}}} - {\text{Abs}}_{{660{\text{ sample}}}} } \right)} \right]}}{{\left( {{\text{Abs}}_{{537{\text{ control}}}} - {\text{Abs}}_{{660{\text{ control}}}} } \right)}}$$

2 $${\text{Selectivity index}} = { }\frac{{{\text{IC}}_{{50{\text{ Vero}}}} }}{{{\text{IC}}_{{50{\text{ HCT}}116}} }}$$

### Statistical analysis

All experiments were performed with at least three replicates (n = 3). Results were presented as mean ± standard deviation. The difference between each group was determined using the non-parametric Kruskal–Wallis test. The statistical analysis was performed using IBM SPSS Statistics software (version 28, SPSS Inc., IL, USA). *p*-values less than 0.05 were considered statistically significant.

## Supplementary Information

Supplementary Information.

## Data Availability

The datasets generated during and/or analyzed during the current study are available from the corresponding author on reasonable request.
