# Supplementary material for: Extracellular vesicles derived from microgreens of Raphanus sativus L. var. caudatus Alef contain bioactive macromolecules and inhibit HCT116 cells proliferation
Source: Sci Rep. 2022 Sep 20;12:15686. doi: 10.1038/s41598-022-19950-7 (PMC9489735; doi:10.1038/s41598-022-19950-7)
Supplement: Supplementary file 1 — Supplementary Information. [file 41598_2022_19950_MOESM1_ESM.docx]

**Supplementary information**

**Extracellular vesicles derived from microgreens of *Raphanus sativus* L. var. *caudatus* Alef contain bioactive macromolecules and inhibit HCT116 cells proliferation**

Karnchanok Kaimuangpak^a^, Kawintra Tamprasit^b^, Kanjana Thumanu^c^, and Natthida Weerapreeyakul^b,d,*^

^a^ Graduate School (in the program of Research and Development in Pharmaceuticals), Faculty of Pharmaceutical Sciences, Khon Kaen University, Khon Kaen, 40002, Thailand

^b^ Research Institute for Human High Performance and Health Promotion, Khon Kaen University, Khon Kaen, 40002, Thailand

^c^ Synchrotron Light Research Institute (Public Organization), Nakhon Ratchasima, 30000, Thailand

^d^ Division of Pharmaceutical Chemistry, Faculty of Pharmaceutical Sciences, Khon Kaen University, Khon Kaen, 40002, Thailand

***Corresponding author:** Associate Professor Natthida Weerapreeyakul Ph.D.,

Division of Pharmaceutical Chemistry, Faculty of Pharmaceutical Sciences, 123 Mittrapap Road, Amphoe Muang, Khon Kaen University, Khon Kaen, 40002, Thailand, Tel: 66**–**43**–**202378, Fax: 66**–**43**–**202379, e-mail: natthida@kku.ac.th

**List of authors’ email addresses**:

Karnchanok Kaimuangpak karkai@kku.ac.th

Kawintra Tamprasit ta.kawintra@kkumail.com

Kanjana Thumanu kanjanat@slri.or.th

*Natthida Weerapreeyakul natthida@kku.ac.th

**Running Title:** EVs from *R. sativus* L. var. *caudatus* Alef microgreens contain biological molecules and inhibit HCT116 colon cancer proliferation

**Table S1** Designated wavenumber (cm^–1^), FTIR spectral band assignment, and referred biocomponents.

| **Wave number**  **(cm^–1^)** | **FTIR spectral band assignment** | **Biomolecules** | **References** |
| --- | --- | --- | --- |
| 3,293 | ν (N–H) of peptide groups, amide A | Proteins | ^50,51,53^ |
| 2,956 and 2,927 | ν_as_ (CH_2_) and ν_as_ (CH_3_) of acyl chain lipids | Lipids | ^50–54^ |
| 2,925 | ν_as_ (CH_2_) of saturated fatty acid | Lipids | ^76^ |
| 2,875 and 2,856 | ν_s_ (CH_2_) and ν_s_ (CH_3_) of acyl chain lipids | Lipids | ^50–54^ |
| 2,852 | ν_s_ (CH_2_) of saturated fatty acid | Lipids | ^88^ |
| 1,741 | ν (C=O) of ester from phospholipids, triglycerides, and cholesterols | Lipids | ^51–54^ |
| 1,656 | ν (C=O) of the peptide backbone,  amide I | Proteins | ^50–54^ |
| 1,546 | δ (N–H) of peptides, amide II | Proteins | ^50–54^ |
| 1,450 | δ_as_ (CH_3_) of lipid acyl chains | Lipids | ^50,51^ |
| 1,406 | δ (CH_3_) of acyl residues from lipids and proteins | Lipids and proteins | ^50,51,54^ |
| 1,309 and 1,247 | ν (C–N) of proteins, amide III | Proteins | ^50^ |
| 1,247 | ν_as_ (PO_2_) of RNA | Nucleic acids | ^57^ |
| 1,226 | ν_as_ (PO_2_) of DNA | Nucleic acids | ^57,89^ |
| 1,154 | ν (C–O) and δ (C–O–H) of carbohydrates | Carbohydrates | ^90^ |
| 1,114 | ν_as_ (PO_2_) of RNA and ν (–OH) at C2′ of ribose in RNA1 | Carbohydrates | ^57^ |
| 1,077 | ν_s_ (PO_2_) of DNA and RNA | Nucleic acids | ^54^ |
| 1,057 | ν (C–O) of polysaccharides | Carbohydrates | ^91^ |
| 1,028 | ν (C–O) and δ (C–OH) of oligosaccharides and polysaccharides | Carbohydrates | ^90^ |

ν = stretching vibration, ν_as_ = asymmetrical stretching vibration, ν_s_ = symmetrical stretching vibration, and δ = bending vibration.

**Table S2** Retention time of sulforaphene (SE), sulforaphane (SF), and their content in the microgreen extract, and EVs.

| **No.** | **Sample** | **Retention time**  **(min)** | | **ITCs content**  **(mg/g fresh weight)** | |
| --- | --- | --- | --- | --- | --- |
|  |  | **SE** | **SF** | **SE** | **SF** |
| 1 | Standard sulforaphene (SE) | 23.50 ± 0.005 | – | – | – |
| 2 | Standard sulforaphane (SF) | – | 26.37 ± 0.001 | – | – |
| 3 | DCM crude extract | 23.47 ± 0.006 | N.D. | 6.09 ± 0.22 | N.D. |
| 4 | EVs | N.D. | N.D. | N.D. | N.D. |

N.D. = Not detected. Data are presented as the mean ± SD (n = 3).

**Table S3** Summary of the statical analysis. The statistical analyses of anti-proliferative activity of EVs, microgreen extract, and cisplatin in HCT116 and Vero cell lines at 24 and 48 hr were performed using the nonparametric independent samples Kruskal–Wallis one–way ANOVA test with the multiple comparison in stepwise stepdown as a post hoc analysis.

| **Groups** | **Total N** | **Test statistic^a^** | **Degree**  **of freedom** | **Asymptotic sig.**  **(2-sided test)^b^** |
| --- | --- | --- | --- | --- |
| EVs-HCT116 (24 and 48 hr) | 48 | 37.684 | 11 | <0.001 |
| EVs-Vero (24 and 48 hr) | 36 | 32.337 | 11 | <0.001 |
| Microgreen extract-HCT116 (24 and 48 hr) | 60 | 45.573 | 11 | <0.001 |
| Microgreen extract-Vero (24 and 48 hr) | 36 | 30.904 | 11 | 0.001 |
| Cisplatin-HCT116 (24 and 48 hr) | 40 | 36.110 | 11 | <0.001 |
| Cisplatin-Vero (24 and 48 hr) | 36 | 33.301 | 11 | <0.001 |

a. The test statistic is adjusted for ties.

b. The significance level is 0.050.

**Table S4** Multiple comparison in stepwise stepdown post–hoc test of anti-proliferative activity of EVs in HCT116 cell line at 24 and 48 hr following the the nonparametric independent samples Kruskal–Wallis one–way ANOVA test, showing 5 homogeneous subsets.

| **Statistical values** | | **Subset^a^** | | | | |
| --- | --- | --- | --- | --- | --- | --- |
|  |  | **1** | **2** | **3** | **4** | **5** |
| Sample average rank | EVs_HCT116_48hr_1000µg/ml | 2.000 |  |  |  |  |
|  | EVs_HCT116_48hr_500µg/ml |  | 5.000 |  |  |  |
|  | EVs_HCT116_24hr_1000µg/ml |  |  | 9.900 |  |  |
|  | EVs_HCT116_48hr_250µg/ml |  |  | 14.667 |  |  |
|  | EVs_HCT116_48hr_62.5µg/ml |  |  | 15.500 | 15.500 |  |
|  | EVs_HCT116_48hr_125µg/ml |  |  | 17.667 | 17.667 | 17.667 |
|  | EVs_HCT116_24hr_500µg/ml |  |  |  | 30.400 | 30.400 |
|  | EVs_HCT116_48hr_Ctrl |  |  |  |  | 31.500 |
|  | EVs_HCT116_24hr_Ctrl |  |  |  |  | 33.000 |
|  | EVs_HCT116_24hr_250µg/ml |  |  |  |  | 34.100 |
|  | EVs_HCT116_24hr_125µg/ml |  |  |  |  | 36.600 |
|  | EVs_HCT116_24hr_62.5µg/ml |  |  |  |  | 39.400 |
| Test Statistic | | .^b^ | .^b^ | 6.355 | 5.648 | 10.253 |
| Sig. (2-sided test) | | . | . | .096 | .059 | .114 |
| Adjusted Sig. (2-sided test) | | . | . | .260 | .217 | .188 |

a. Homogeneous subsets are based on asymptotic significances. The significance level is 0.050.

b. Unable to compute because the subset contains only one sample.

**Table S5** Multiple comparison in stepwise stepdown post–hoc test of anti-proliferative activity of EVs in Vero cell line at 24 and 48 hr following the the nonparametric independent samples Kruskal–Wallis one–way ANOVA test, showing 5 homogeneous subsets.

| **Statistical values** | | **Subset^a^** | | | | |
| --- | --- | --- | --- | --- | --- | --- |
|  |  | **1** | **2** | **3** | **4** | **5** |
| Sample average rank | EVs_Vero_48hr_1000µg/ml | 2.000 |  |  |  |  |
|  | EVs_Vero_48hr_500µg/ml | 6.000 | 6.000 |  |  |  |
|  | EVs_Vero_48hr_125µg/ml | 8.000 | 8.000 | 8.000 |  |  |
|  | EVs_Vero_48hr_250µg/ml |  | 10.000 | 10.000 |  |  |
|  | EVs_Vero_24hr_1000µg/ml |  |  | 14.000 |  |  |
|  | EVs_Vero_48hr_62.5µg/ml |  |  |  | 17.000 |  |
|  | EVs_Vero_24hr_125µg/ml |  |  |  | 23.667 | 23.667 |
|  | EVs_Vero_48hr_Ctrl |  |  |  | 24.333 | 24.333 |
|  | EVs_Vero_24hr_Ctrl |  |  |  | 24.333 | 24.333 |
|  | EVs_Vero_24hr_500µg/ml |  |  |  | 28.667 | 28.667 |
|  | EVs_Vero_24hr_250µg/ml |  |  |  | 31.667 | 31.667 |
|  | EVs_Vero_24hr_62.5µg/ml |  |  |  |  | 32.333 |
| Test Statistic | | 5.647 | 3.227 | 5.600 | 10.942 | 8.088 |
| Sig. (2-sided test) | | .059 | .199 | .061 | .053 | .151 |
| Adjusted Sig. (2-sided test) | | .217 | .589 | .222 | .102 | .280 |

a. Homogeneous subsets are based on asymptotic significances. The significance level is 0.050.

**Table S6** Multiple comparison in stepwise stepdown post–hoc test of anti-proliferative activity of microgreen extract in HCT116 cell line at 24 and 48 hr following the the nonparametric independent samples Kruskal–Wallis one–way ANOVA test, showing 6 homogeneous subsets.

| **Statistical values** | | **Subset^a^** | | | | | |
| --- | --- | --- | --- | --- | --- | --- | --- |
|  |  | **1** | **2** | **3** | **4** | **5** | **6** |
| Sample average rank | Micro_HCT116_48hr_250µg/ml | 3.000 |  |  |  |  |  |
|  | Micro_HCT116_24hr_250µg/ml |  | 8.200 |  |  |  |  |
|  | Micro_HCT116_48hr_125µg/ml |  |  | 14.800 |  |  |  |
|  | Micro_HCT116_48hr_31.25µg/ml |  |  | 28.400 | 28.400 |  |  |
|  | Micro_HCT116_24hr_Ctrl |  |  | 29.300 | 29.300 | 29.300 |  |
|  | Micro_HCT116_48hr_62.5µg/ml |  |  | 30.000 | 30.000 | 30.000 |  |
|  | Micro_HCT116_48hr_15.625µg/ml |  |  | 30.500 | 30.500 | 30.500 |  |
|  | Micro_HCT116_48hr_Ctrl |  |  | 31.300 | 31.300 | 31.300 |  |
|  | Micro_HCT116_24hr_125µg/ml |  |  | 41.000 | 41.000 | 41.000 | 41.000 |
|  | Micro_HCT116_24hr_31.25µg/ml |  |  |  | 46.200 | 46.200 | 46.200 |
|  | Micro_HCT116_24hr_62.5µg/ml |  |  |  |  | 48.400 | 48.400 |
|  | Micro_HCT116_24hr_15.625µg/ml |  |  |  |  |  | 54.900 |
| Test Statistic | | .^b^ | .^b^ | 12.056 | 8.887 | 12.441 | 4.409 |
| Sig. (2-sided test) | | . | . | .061 | .180 | .053 | .221 |
| Adjusted Sig. (2-sided test) | | . | . | .102 | .288 | .089 | .526 |

a. Homogeneous subsets are based on asymptotic significances. The significance level is 0.050.

b. Unable to compute because the subset contains only one sample.

**Table S7** Multiple comparison in stepwise stepdown post–hoc test of anti-proliferative activity of microgreen extract in Vero cell line at 24 and 48 hr following the the nonparametric independent samples Kruskal–Wallis one–way ANOVA test, showing 6 homogeneous subsets.

| **Statistical values** | | **Subset^a^** | | | | | |
| --- | --- | --- | --- | --- | --- | --- | --- |
|  |  | **1** | **2** | **3** | **4** | **5** | **6** |
| Sample average rank | Micro_Vero_48hr_250µg/ml | 3.333 |  |  |  |  |  |
|  | Micro_Vero_24hr_250µg/ml | 3.667 | 3.667 |  |  |  |  |
|  | Micro_Vero_48hr_125µg/ml | 9.000 | 9.000 | 9.000 |  |  |  |
|  | Micro_Vero_24hr_125µg/ml |  | 10.000 | 10.000 | 10.000 |  |  |
|  | Micro_Vero_48hr_62.5µg/ml |  |  | 15.333 | 15.333 | 15.333 |  |
|  | Micro_Vero_48hr_31.25µg/ml |  |  |  | 19.000 | 19.000 | 19.000 |
|  | Micro_Vero_48hr_15.625µg/ml |  |  |  | 20.333 | 20.333 | 20.333 |
|  | Micro_Vero_24hr_62.5µg/ml |  |  |  | 22.667 | 22.667 | 22.667 |
|  | Micro_Vero_24hr_Ctrl |  |  |  |  | 29.000 | 29.000 |
|  | Micro_Vero_48hr_Ctrl |  |  |  |  | 29.333 | 29.333 |
|  | Micro_Vero_24hr_31.25µg/ml |  |  |  |  |  | 30.000 |
|  | Micro_Vero_24hr_15.625µg/ml |  |  |  |  |  | 30.333 |
| Test Statistic | | 5.422 | 5.600 | 5.600 | 8.833 | 10.544 | 10.459 |
| Sig. (2-sided test) | | .066 | .061 | .061 | .065 | .061 | .107 |
| Adjusted Sig. (2-sided test) | | .241 | .222 | .222 | .150 | .119 | .176 |

a. Homogeneous subsets are based on asymptotic significances. The significance level is 0.050.

**Table S8** Multiple comparison in stepwise stepdown post–hoc test of anti-proliferative activity of cisplatin in HCT116 cell line at 24 and 48 hr following the the nonparametric independent samples Kruskal–Wallis one–way ANOVA test, showing 7 homogeneous subsets.

| **Statistical values** | | **Subset^a^** | | | | | | |
| --- | --- | --- | --- | --- | --- | --- | --- | --- |
|  |  | **1** | **2** | **3** | **4** | **5** | **6** | **7** |
| Sample average rank | Cis_HCT116_48hr_120µg/ml | 2.000 |  |  |  |  |  |  |
|  | Cis_HCT116_48hr_60µg/ml |  | 5.333 |  |  |  |  |  |
|  | Cis_HCT116_24hr_120µg/ml |  | 7.667 |  |  |  |  |  |
|  | Cis_HCT116_48hr_30µg/ml |  |  | 11.000 |  |  |  |  |
|  | Cis_HCT116_24hr_60µg/ml |  |  |  | 14.000 |  |  |  |
|  | Cis_HCT116_24hr_30µg/ml |  |  |  |  | 17.333 |  |  |
|  | Cis_HCT116_48hr_15µg/ml |  |  |  |  | 20.333 | 20.333 |  |
|  | Cis_HCT116_24hr_15µg/ml |  |  |  |  | 24.333 | 24.333 | 24.333 |
|  | Cis_HCT116_24hr_7.5µg/ml |  |  |  |  |  | 28.167 | 28.167 |
|  | Cis_HCT116_48hr_Ctrl |  |  |  |  |  | 31.900 | 31.900 |
|  | Cis_HCT116_24hr_Ctrl |  |  |  |  |  |  | 32.500 |
|  | Cis_HCT116_48hr_7.5µg/ml |  |  |  |  |  |  | 35.833 |
| Test Statistic | | .^b^ | 2.333 | .^b^ | .^b^ | 5.067 | 7.712 | 6.879 |
| Sig. (2-sided test) | | . | .127 | . | . | .079 | .052 | .142 |
| Adjusted Sig. (2-sided test) | | . | .556 | . | . | .282 | .149 | .308 |

a. Homogeneous subsets are based on asymptotic significances. The significance level is 0.050.

b. Unable to compute because the subset contains only one sample.

**Table S9** Multiple comparison in stepwise stepdown post–hoc test of anti-proliferative activity of cisplatin in Vero cell line at 24 and 48 hr following the the nonparametric independent samples Kruskal–Wallis one–way ANOVA test, showing 6 homogeneous subsets.

| **Statistical values** | | **Subset^a^** | | | | | |
| --- | --- | --- | --- | --- | --- | --- | --- |
|  |  | **1** | **2** | **3** | **4** | **5** | **6** |
| Sample average rank | Cis_Vero_48hr_60µg/ml | 5.333 |  |  |  |  |  |
|  | Cis_Vero_48hr_120µg/ml | 5.667 |  |  |  |  |  |
|  | Cis_Vero_48hr_30µg/ml | 7.000 |  |  |  |  |  |
|  | Cis_Vero_24hr_120µg/ml | 8.000 | 8.000 |  |  |  |  |
|  | Cis_Vero_24hr_60µg/ml | 15.333 | 15.333 | 15.333 |  |  |  |
|  | Cis_Vero_48hr_15µg/ml |  | 15.667 | 15.667 |  |  |  |
|  | Cis_Vero_48hr_7.5µg/ml |  |  | 20.333 | 20.333 |  |  |
|  | Cis_Vero_24hr_30µg/ml |  |  |  | 23.667 | 23.667 |  |
|  | Cis_Vero_24hr_15µg/ml |  |  |  | 25.000 | 25.000 |  |
|  | Cis_Vero_24hr_7.5µg/ml |  |  |  |  | 29.000 | 29.000 |
|  | Cis_Vero_48hr_Ctrl |  |  |  |  |  | 33.000 |
|  | Cis_Vero_24hr_Ctrl |  |  |  |  |  | 34.000 |
| Test Statistic | | 7.473 | 5.468 | 5.422 | 4.622 | 5.600 | 5.600 |
| Sig. (2-sided test) | | .113 | .065 | .066 | .099 | .061 | .061 |
| Adjusted Sig. (2-sided test) | | .250 | .236 | .241 | .341 | .222 | .222 |

a. Homogeneous subsets are based on asymptotic significances. The significance level is 0.050.
